# Clinical-Pathological Conference Series from the Medical University of Graz: Case No 178: A 30-year-old nurse with urine dipstick (+++)‑positive for protein in her late pregnancy

**DOI:** 10.1007/s00508-023-02316-y

**Published:** 2024-02-20

**Authors:** Elisabeth Fabian, Gert Mayer, Kathrin Eller, Marion Pollheimer, Robert Queissner, Guenter J. Krejs

**Affiliations:** 1 Department of Internal Medicine II, University Hospital Krems, Karl Landsteiner University of Health Sciences, Krems on the Danube, Austria; 2 Division of Nephrology and Hypertension, Department of Internal Medicine IV, Medical University of Innsbruck, Innsbruck, Austria; 3 Division of Nephrology, Department of Internal Medicine, Medical University of Graz, Graz, Austria; 4 Department of Pathology, Medical University of Graz, Graz, Austria; 5 Department of Psychiatry, Medical University of Graz, Graz, Austria; 6 Division of Gastroenterology and Hepatology, Department of Internal Medicine, Medical University of Graz, Auenbruggerplatz 15, 8036 Graz, Austria

**Keywords:** Factitious disease, Albuminuria, Acute kidney disease, Hyperkalemia, Munchausen syndrome

## Presentation of case

### Dr. K. Eller:

The patient is a nurse working in an intensive care unit in a Styrian hospital. At 32 weeks pregnant (first pregnancy) she presented to the Nephrology Clinic at Graz University Medical Center because of proteinuria (urine dipstick: protein +++). As her general condition was good and there were no clinical signs of nephrotic syndrome (no edema, blood pressure 130/80 mm Hg), she was referred for further diagnostic workup on an outpatient basis.

Laboratory: erythrocytes 4.0 × 10^12^/L (normal: 4.1–5.1 × 10^12^/L), hemoglobin 12.2 g/dL (normal: 12.0–15.3 g/dL), hematocrit 35.3% (normal: 35.0–55.0%), mean corpuscular volume 88.5 fL (normal: 80–98 fL), leukocytes 8.86 × 10^9^/L (normal: 4.4–11.3 × 10^9^/L), platelets 249 × 10^9^/L (normal: 140–440 × 10^9^/L), creatinine 0.7 mg/dL (normal: < 1.0 mg/dL), urea 19 mg/dL (normal: 10–45 mg/dL), uric acid 5.1 mg/dL (normal: 2.4–5.7 mg/dL), estimated glomerular filtration rate (eGFR) 117 mL/min/1.73 m^2^ (normal: 90–120 mL/min/1.73 m^2^), total protein 6.6 g/dL (normal: 6.6–8.3 g/dL), albumin 3.6 g/dL (normal: 3.5–5.3 g/dL). Serum electrolytes (sodium, potassium, chloride, calcium, magnesium, phosphate) and liver parameters were normal, alkaline phosphatase 117 U/L (normal: 35–110 U/L), C‑reactive protein (CRP) 9.7 mg/L (normal: < 5.0 mg/L). Serum glucose, creatine kinase and lactate dehydrogenase were within normal limits. Antinuclear antibodies (ANA) 1:1280. The following were all normal or negative: DFS 70 antibodies, cytoplasmic antibodies, extractable nuclear antigens (ENA) screening, anti-double stranded deoxyribonulceic acid (anti-dsDNA) antibodies, anti-native-DNA against Crithidia (anti-nDNA) antibodies, antibodies against ribosomal protein, myeloperoxidase (MPO)-antineutrophil cytoplasmic antibodies (ANCA), proteinase 3 (Pr3)-ANCA, perinuclear (*p*)-ANCA, cytoplasmic (*c*)-ANCA, basal membrane antibodies, glomerular basal membrane antibodies, antiphospholipase-A2 receptor antibodies and antibodies against THSD7A. Also normal: serum protein electrophoresis, immunoglobulins, kappa light chains, lambda light chains, C3 and C4 complement factors. Urine: total protein 10,778 mg/L (normal: < 130 mg/L), total protein/creatinine 7569 mg/g creatinine (normal: < 110 mg/g creatinine), albumin 11,076 mg/L (normal: 0–20 mg/L), albumin/creatinine 7778 (normal: 0–30 mg/g creatinine), immunoglobulin G < 4 mg/L (normal: < 10 mg/L), α1-microglobulin 15.9 mg/L (normal: < 12 mg/L), α1-microglobulin/creatinine 11 mg/g creatinine (normal: < 16 mg/g creatinine), β2-microglobulin 263 µg/L (normal: < 300 µg/L). Urine sediment examination was without pathologic findings.

Her history was positive for lupus antibodies, factor V Leiden mutation and immune thrombocytopenia, which had been treated with rituximab and eltrombopag. Further, she had undergone flavectomy (L4/L5/S1). There is also a history of obesity (her current body weight is 113 kg and body height 165 cm). For prophylaxis of thromboembolism and preeclampsia the patient received low molecular weight heparin (60 mg s.c. daily) and acetylsalicylic acid (150 mg p.o. daily) during pregnancy.

As the patient’s urine protein loss was again about 20 g per day at the next outpatient visit, and she additionally presented with edema (on both lower extremities), she was admitted for induction of labor. A healthy boy was born by Cesarean section.

A kidney biopsy was scheduled 1 month after delivery because the patient’s urine protein levels were still elevated; however, it could not be performed because her aPTT was prolonged at 95 s (normal: 26–36 s) on that day. The coagulation parameters 6 weeks later were back to normal and renal biopsy sample was successfully obtained. About 6 h after the intervention, the patient started bleeding with hemodynamic instability for which she received four units of packed red blood cells. Computed tomography of the abdomen showed a retroperitoneal hematoma of 9 × 7 cm. Finally, the postinterventional bleeding was managed with coil embolization of the lower segment of the left renal artery. Histology was unremarkable; podocytes were intact on electron microscopy.

At the next outpatient visit, her urine protein level increased to 34 g per day. Treatment with corticosteroids was instituted. In the further course creatinine (1.5 mg/dL) and potassium (6.3 mmol/L) levels markedly increased. At a serum potassium value of 8 mmol/L, the patient was admitted to the intensive care unit.

A diagnostic measure was performed.

## Differential diagnosis

### Dr. G. Mayer:

This is an interesting case of a young woman with proteinuria on dipstick examination in her late pregnancy.

Proteinuria is defined as increased protein excretion in the urine (greater than 150–160 mg/24 h in adults) and is a sign of an underlying renal abnormality, usually glomerular in origin when greater than 1 g/day [1]. Kidney disease usually presents in one of two ways: as an incidental finding during a routine medical evaluation or with evidence of renal dysfunction, such as hypertension, edema and hematuria. In both situations, the assessment of the cause and severity of renal abnormality is pivotal. Urinalysis is an important diagnostic tool and includes dipstick examination, followed by microscopic assessment if the dipstick is positive [1]. The dipstick test measures urine pH, protein, hemoglobin, glucose, ketones, bilirubin, nitrites and leukocytes; however, regarding proteinuria, one should keep in mind that urine dipstick tests detect mostly albumin and give false positive results when pH > 7.0 or when the urine is very concentrated or contaminated by blood. A very dilute urine may obscure significant proteinuria on dipstick test, and proteinuria that is not predominantly albuminuria will be missed [2]. Thus, whenever the urine dipstick is positive, a detailed laboratory analysis and microscopic assessment have to be performed. In this case, it was peculiar that proteinuria and albuminuria were more or less identical (7.6 g protein/g creatinine; 7.8 g albumin/g creatinine). Moreover, of note is that creatinine and eGFR as well as serum levels of total protein and albumin were within normal limits, whereas renal protein and albumin excretion were markedly increased. The amount of protein loss in the urine and its composition depend on the underlying renal injury. Both the charge and size selectivity normally prevent virtually all plasma albumin, globulins and other large molecular weight proteins from crossing the glomerular wall; however, when this barrier is disrupted there can be leakage of plasma proteins into urine [2]. There are different forms of proteinuria depending on the underlying pathomechanism: (1) glomerular proteinuria results from effacement of epithelial cell foot processes (podocytes) and altered glomerular permeability with an increased filtration fraction of normal plasma proteins [1]. Some glomerular diseases, such as minimal change disease, cause fusion of glomerular epithelial cell foot processes, resulting in predominantly “selective” loss of albumin. Other glomerular diseases can present with disruption of the basement membrane and slit diaphragms (e.g., by immune complex deposition), leading to marked loss of albumin and other plasma proteins. The fusion of foot processes causes increased flow and pressure across the capillary basement membrane, which results in areas with larger pore sizes. The combination of increased pressure and larger pores results in significant “nonselective” proteinuria [2]. (2) Tubular proteinuria occurs as a result of faulty reabsorption of freely filtered smaller proteins (< 20 kDa; e.g., β2 microglobulin, apoproteins, enzymes and peptide hormones) by the proximal tubule. (3) Overload proteinuria is due to excessive production of abnormal filterable plasma proteins (e.g., Bence-Jones proteins associated with multiple myeloma) that exceeds the reabsorption capacity of the tubule [1, 2]. Thus, the pattern of proteinuria usually helps to identify the underlying cause of protein loss.

Given a selective albuminuria in this patient, the differential list includes kidney diseases, such as diabetic nephropathy, membranous lupus nephritis (class V), lupus podocytopathy, minimal change nephritis and early-onset focal segmental glomerulosclerosis; however, a normal kidney biopsy as documented here rules out these diseases. Furthermore, preeclampsia or HELLP (Hemolysis, Elevated Liver enzymes and Low Platelets) syndrome can also be taken off the differential list because, except for proteinuria, the patient did not show symptoms such as new-onset hypertension, significant end-organ dysfunction or laboratory findings such as thrombocytopenia and increased liver enzymes.

This leads to the hypothesis that albumin was not lost in the kidneys but rather that it entered urine somehow afterwards. As the discussed patient is a nurse, and as such has unlimited access to various medications including human albumin, factitious disease should be considered as a differential diagnosis in this case. The pathologic conscious and voluntary production of physical diseases can be found in all medical disciplines and is often unrecognized or misdiagnosed. Factitious disorder is often associated with unusual symptom presentation, self-inflicted injuries, unnecessary invasive interventions, unusual and protracted recovery and a frequent switching of treating physicians [3]. In this case, the patient may have added albumin to her excreted urine and faked albuminuria.

Another mechanism that may explain selective albuminuria in a patient with normal kidney biopsy is chyluria. This rare condition is secondary to the presence of chyle in the urine due to a fistulous communication between the lymphatic system and the urine tract [4, 5]. About 70% of cases with chyluria present with milky white urine, but this is not observed in all affected patients [6]. Chyluria can be broadly classified into parasitic and non-parasitic causes. Worldwide, about 95% of parasitic causes are attributable to *Wuchereria bancrofti*; others are due to echinococcosis, hymenolepiasis, ankylostomiasis, trichiniosis and malaria. Nonparasitic causes are rare and include blunt or penetrating trauma, a complication of surgery such as partial nephrectomy or aortoiliac bypass, infection, malignancy, lymphatic malformation, radiation, abscess, congenital abnormalities, pregnancy and stenosis of the thoracic duct [5, 7–11]. Previous spinal surgery (flavectomy, L4/L5/S1) and pregnancy are two factors that may have predisposed this patient to the development of chyluria and concomitant albuminuria; however, in this case one would expect recovery of albuminuria after delivery, but the contrary was observed: albuminuria and also renal function parameters and serum potassium increased markedly, although with significant discrepancy (serum creatinine 1.5 mg/dL; serum potassium 8.0 mmol/L). Her positive history for lupus antibodies and factor V Leiden mutation, and increased levels of CRP and ANA suggest an association with the antiphospholipid syndrome and an overall prothrombogenic situation that may have caused renal vein thrombosis subsequently leading to acute kidney injury and hyperkalemia secondary to primary hypoaldosteronism due to thrombosis of the adrenal vein. This is based on the presumption that prophylactic anticoagulation with low molecular weight heparin was discontinued when the patient presented with bleeding after biopsy. The presence of a thrombosis should be confirmed by magnetic resonance venography or computed tomography angiography.

## Dr. G. Mayer’s diagnosis

Chyluria, antiphospholipid syndrome, thrombosis of renal and adrenal veins

## Discussion of case

### Dr. K. Eller:

**Fig. 1:**
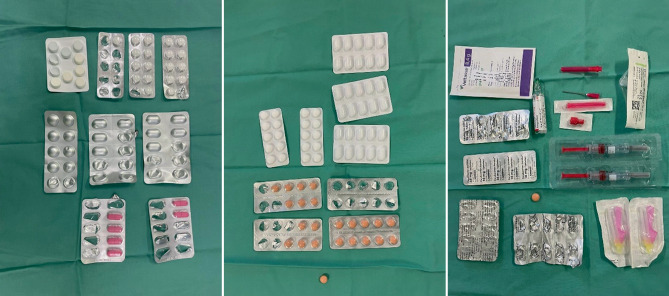
Various drugs (diuretics, antihypertensives, NSAIDs, anticoagulants) found on room search

As summarized by Dr. Mayer, this case of proteinuria was really challenging because of various discrepancies between the history, clinical presentation, histology and laboratory data; however, soon after admission it became clear that the patient was faking kidney disease, i.e., albuminuria, acute renal failure and hyperkalemia by adding human albumin to her urine and abusing diuretics (loop diuretics and aldosterone antagonists), antihypertensives (ACE inhibitor, AT2 blocker and urapidil), NSAIDs and KCl tablets. All these drugs were found in the patient’s handbag on a room search (Fig. 1). Furthermore, urinalysis clearly showed misuse of diuretics (Table 1). Moreover, we found acetylsalicylic acid tablets and low molecular weight heparin for subcutaneous application in the patient’s handbag, which she had administered herself (multiple puncture sites were found on her body) and we speculated that these drugs might have led to the postinterventional bleeding after the renal biopsy. In the intensive care unit, urine obtained by a urine catheter was negative for protein. Confronted with these findings and the laboratory data, the patient admitted to faking kidney disease, which therefore confirmed the diagnosis of factitious disease. A psychiatrist was consulted.

**Table 1:** Levels of diuretics in urine of the discussed patient

Drug	Concentration (ng/mL)	Limit of detection (ng/mL)
Canrenone (metabolite of spironolactone)	3,800	1.0
Eplerenone	9,600	1.0
Furosemide	32,000	5.0

Drug measurements were performed in the laboratory of Dr. Guenter Gmeiner, Seibersdorf Labor GmbH, Seibersdorf, Austria. This laboratory is the reference laboratory for doping in Austria. The analysis serves the detection of the presence of drugs in urine. Concentrations of the drugs in urine during normal therapeutic use have not and cannot be established (they exist only for concentrations in serum or blood)

### Dr. G.J. Krejs:

Factitious disorder imposed on self (Munchausen syndrome) is defined as the “intentional production or feigning of symptoms or disabilities, either physical or psychological” [12]. Individuals assuming the role of a patient by manifesting physical or psychological symptoms without conscious or obvious reward are encountered in all disciplines of clinical medicine [13]. Diagnosis of factitious disease is often difficult because of its many forms and various degrees of severity and many cases probably go unrecognized. Data about the frequency of factitious disease are scarcely available, but it is estimated that the 1‑year prevalence ranges from about 1% to 5% [14–18]. About 90% of patients produce symptoms in a self-harming manner [3]. As in the discussed case, the majority of affected patients are typically female in the mid-thirties with a healthcare or laboratory profession [3, 18]. In the 36-year history of clinical-pathological conferences at the Medical University of Graz, this is the fifth such case (5 out of 178). Besides the current case, these included patients feigning coagulopathy by surreptitious intake of warfarin, hypoglycemia due to self-injection of insulin, diarrhea caused by surreptitious laxative abuse [19] and repeated induction of abscesses by self-injection of stool into the abdominal wall.

Although patients with factitious disease may appear in any specialist setting, internal medicine (endocrinology, cardiology, gastroenterology, nephrology), dermatology, neurology and emergency departments tend to be more often confronted with such patients [18, 20].

In the majority of cases an unsubstantiated presentation (78%), past healthcare service use leading to a large amount of medical records (47%), atypical presentation (40%), treatment failure (35%), investigations indicating fabrication (33%), patient behavior (31%) or evidence of fabrication (31%) contribute to the diagnosis of factitious disease [18]. Between 40% and 64% of cases remain suspected [3].

Hints from the laboratory sometimes help to identify patients with factitious disease. For example, in hypoglycemia induced by self-injection of insulin, laboratory analysis will show a triad of hypoglycemia, high immunoreactive insulin and normal or suppressed plasma C‑peptide immunoreactivity. When sulfonylureas, repaglinide or nateglinide are the suspected cause of hypoglycemia, a plasma level analysis of these drugs is required to distinguish laboratory findings from those of insulinoma [1]. In exogenous hyperthyroidism, which is caused by the intake of excess amounts of thyroid hormones, laboratory data will show a low serum level of thyroglobulin (by contrast, it is high in patients with endogenous hyperthyroidism) and there is hardly any 24‑h radioiodine uptake in the thyroid due to the suppression of TSH secretion (except in patients with some autonomously functioning thyroid tissue) [21]. In cases of factitious proteinuria fabricated by adding human albumin to the patient’s urine, performance of sodium dodecyl sulfate polyacrylamide gel electrophoresis (SDS-PAGE) of urine proteins will be helpful. In contrast to real proteinuria, SDS-PAGE will detect only an albumin but no transferrin band when proteinuria is feigned. To rule out the rare condition of congenital atransferrinemia as the underlying reason for the missing transferrin band, electrophoresis may also be performed in serum [22]. These examples emphasize that it may be useful to perform certain specific laboratory tests in order to identify self-induced symptoms. Furthermore, a room search often helps to find evidence of fabrication and to confront the patient. When a specific drug is found during a room search, the methods of forensic medicine can be employed to prove its presence or the presence of its metabolites in serum or urine; however, diagnosing factitious disease is often challenging and generally rests on supportive rather than conclusive evidence. The danger of misdiagnosis and inappropriate treatment is high [3].

Studies of factitious disease demonstrate the heavy impact of unnecessary investigations, treatment and hospital admissions on the healthcare system. Repeated inpatient stays of up to 25 days are frequently observed [20, 23]. In the USA, factitious diseases are estimated to account for about $ 20 billion per year in healthcare costs [24]. In individual cases, costs have also exceeded $ 200,000 [25] and even $ 1,000,000 [26].

Factitious disease is a dangerous condition because of the potential of self-harm. In our case, the patient injected heparin and provoked the complication of bleeding following renal biopsy. The senior author has seen cases of factitious disease who have undergone unnecessary colectomy and hemipancreatectomy.

### Dr. R. Queissner:

Factitious disease is a psychiatric disorder in which sufferers intentionally fabricate illness, injury or impairment in order to be admitted and to undergo medical procedures, without any obvious gain [18, 27]. The motivations of patients with factitious disease are “almost always obscure” and may include a desire to receive affection and care, an “adrenaline rush” from undergoing medical procedures or a sense of control from deceiving healthcare professionals [18, 28]. In addition, the fact that factitious actions are strongly tabooed makes it more attractive to affected patients [14, 24, 29].

One can suppose that this disorder occurs more frequently than generally assumed and displays varying degrees of severity. Due to the diversity of clinical presentation, factitious disease is considered to be one of the most challenging disorders in medical practice [30]. The etiopathogenesis of factitious disease is widely unknown but according to psychodynamic developmental psychological and trauma psychological models, objectification and manipulation of the own body and assuming the sick role are attempts to solve subconscious needs and conflicts [3, 31–33]. Mental and behavioral comorbidities such as depression or personality disorders, addiction, eating and stress-related disorders are found in up to 70% of affected patients [18, 34, 35].

Although about 10–30% of factitious acts are isolated and harmless events, episodic or chronic courses appear to be more common [14, 15, 17, 18, 35]. In some cases, factitious behavior can also take on the character of a true addiction [3]. Factitious disease is associated with a low suicide risk [18]; nevertheless, patients may expose themselves to a considerable risk by self-induction of disease. Fatality is rare but may be due to failure to recognize feigning and symptom fabrication which leads to worsening of the prognosis or it may be due to complications from (provoked) interventions [3, 36–40].

As in the discussed patient, the staged drama of suffering, then the injustice and finally disillusionment are typical of factitious disease. Confrontation with the diagnosis can bring considerable relief for patients as their deception is associated with privation and pain. In this case, a stepwise supportive confrontation can also be used to openly explain therapeutic options such as psychotherapy; however, the majority of affected patients will deny feigning illness and confrontation is likely to result in patients “going underground” and continuing their deception in a more differentiated manner in other institutions [3]. Krahn et al. [15] have shown that of 93 patients with factitious disease only 16 admitted feigning illness and only a small number agreed to psychiatric treatment.

Factitious disorders are an important item on the differential diagnosis list but are often unrecognized and misdiagnosed. As the volitional aspect, i.e., perception of deliberate deception in affected patients is blurred, differentiation from functional, dissociative and somatoform disorders and simulation/aggravation is often challenging [3, 41]. The absence of clear external incentives and a strong propensity to self-harm distinguish factitious disease from malingering [3]. A subtype of factitious disease is Munchausen by proxy, i.e., fabrication of symptoms in other persons, usually children or dependents [12, 42].

### Dr. G.J. Krejs:

As far as Munchausen by proxy is concerned, the senior author (when working at the University of Texas Health Science Center at Dallas) had seen a case of a mother fabricating symptoms in her child. She came from Mississippi to the center in Dallas with her 7‑year-old son on a central line for rehydration and electrolyte substitution. During the clinical workup it could be proved that she was causing watery diarrhea in the child by giving multiple daily doses of magnesium sulfate.

### Dr. G. Mayer:

This is an instructive case of factitious disease which is one of the most challenging disorders in medical practice. Patients with factitious disease may fabricate medical needs in several ways. The variety of self-induced symptoms depends not only on the methods available and the patient’s medical knowledge but also on the level of dedication and imagination. I remember another patient who presented several times in our outpatient clinic with laboratory signs compatible with acute kidney disease (massively elevated creatinine and blood urea nitrogen levels) but otherwise with completely normal findings. It turned out that she was drinking massive amounts of her own urine before she went to the hospital.

Clinicians should be aware of factitious disease whenever there are warning signs such as unusual clinical findings, implausible or unusual test results, contradictory laboratory results, worsening of the condition prior to discharge, or worsening or improvement as predicted by the patient [3].

### Dr. K. Eller:

In follow-up, it needs to be reported that the patient returned to another Graz hospital 3 months later and said she wanted to see a nephrologist due to high amounts of protein in her urine; however, a resident, who was on rotation in the Division of Nephrology of Graz University Medical Center while the patient was there, recognized her. Once confronted, the patient left immediately.

## Final diagnosis

Factitious disease (fabrication of proteinuria by addition of human albumin to urine and induction of acute kidney disease and hyperkalemia by surreptitious intake of several drugs)
